# METHODOLOGICAL CHALLENGES AND OPPORTUNITIES FOR STUDYING RESILIENCE

**DOI:** 10.1093/geroni/igad104.1822

**Published:** 2023-12-21

**Authors:** Ravi Varadhan, Karen Bandeen-Roche, George Kuchel

## Abstract

Many older adults experience a major stressor at some point in their lives. The ability to recover and live well after a major stressor is known as resilience. An important goal of geriatric research is to identify factors which influence resilience to stressors, and ultimately, to devise strategies to enhance the resilience of older adults. In order to accomplish these worthy aims, several methodological challenges need to be addressed. Methodologies for studying resilience in clinical settings are only recently being developed. Investigators at Duke, Johns Hopkins, and Radboud Universities are studying cohorts of older individuals who are undergoing invasive clinical procedures using longitudinal designs. These studies collect a vast array of information on each participant including socio-demographics, health status, physical, cognitive, psychological, and biological functions. Also, large amount of experimental data is collected on the dynamics of select physiological systems as probes of resilience (“deep phenotyping”). Deep phenotyping leads to several challenges in the conduct and analysis of the study including selective participation (selection bias), informatively missing data, characterizing resilient responses based upon trajectories of multidimensional measures before and after stressor, modeling dynamical systems experimental data, and quantification of resilience capacity and its determinants. In this symposium, we will discuss some of the critical methodological challenges of studying resilience and present approaches to address them. At the end of this symposium, the audience members should understand the importance of designs and analytic challenges in studying resilience, and become knowledgeable on the approaches to overcome methodological challenges.

